# Diagnostic Biomarkers and Immune Infiltration in Patients With T Cell-Mediated Rejection After Kidney Transplantation

**DOI:** 10.3389/fimmu.2021.774321

**Published:** 2022-01-04

**Authors:** Hai Zhou, Hongcheng Lu, Li Sun, Zijie Wang, Ming Zheng, Zhou Hang, Dongliang Zhang, Ruoyun Tan, Min Gu

**Affiliations:** ^1^Department of Urology, The Second Affiliated Hospital of Nanjing Medical University, Nanjing, China; ^2^Department of Urology, The First Affiliated Hospital of Nanjing Medical University, Nanjing, China

**Keywords:** kidney transplantation, T cell-mediated rejection, diagnostic biomarkers, immune infiltration, bioinformatic analysis

## Abstract

T cell-mediated rejection (TCMR) is an important rejection type in kidney transplantation, characterized by T cells and macrophages infiltration. The application of bioinformatic analysis in genomic research has been widely used. In the present study, Microarray data was analyzed to identify the potential diagnostic markers of TCMR in kidney transplantation. Cell-type identification by estimating relative subsets of RNA transcript (CIBERSORT) was performed to determine the distribution of immune cell infiltration in the pathology. Totally 129 upregulated differently expressed genes (DEGs) and 378 downregulated DEGs were identified. The GO and KEGG results demonstrated that DEGs were mainly associated with pathways and diseases involved in immune response. The intersection of the two algorithms (PPI network and LASSO) contains three overlapping genes (CXCR6, CXCL13 and FCGR1A). After verification in GSE69677, only CXCR6 and CXCL13 were selected. Immune cells Infiltration analysis demonstrated that CXCR6 and CXCL13 were positively correlated with gamma delta T cells (*p* < 0.001), CD4^+^ memory activated T cells (*p* < 0.001), CD8^+^ T cells (*p* < 0.001) and M1 macrophages (*p* = 0.006), and negatively correlated with M2 macrophages (*p* < 0.001) and regulatory T cells (*p* < 0.001). Immunohistochemical staining and image analysis confirmed the overexpression of CXCR6 and CXCL13 in human allograft TCMR samples. CXCR6 and CXCL13 could be diagnostic biomarkers of TCMR and potential targets for immunotherapy in patients with TCMR.

## Introduction

Kidney transplantation is the optimal therapy for end-stage renal disease (1). It improves the quality of life and brings better survival advantages to patients compared to dialysis. Although the risk of graft failure is reducing every year, long-term survival remains suboptimal (2). Rejection is one of the most common cause of allograft loss after transplantation. Rejection in first year post transplant is associated with worse long-term graft outcomes (3). T lymphocytes are the principal cells that recognize the allograft. T cell-mediated rejection (TCMR) is an important event in kidney transplantation and a model for T cell mediated inflammatory diseases (4). Despite the rates of TCMR decreasing for major advances in immunosuppression, the role of genes and immune cells in the occurrence and development of TCMR is still not clear enough.

Recently, bioinformatic analysis has been generally performed in the identification of biomarkers, which provides insight into the molecular mechanism of disease progression (5–9). Microarray technology can be used for a wide range of bioinformatic analysis and the development of new computational techniques remains an active area of bioinformatics research that will continue to expand over the coming years (10–12). Moreover, researches have shown that immune cell infiltration plays an increasingly remarkable role not only in transplantation, but also in various diseases (13–15). However, so far, few studies have applied CIBERSORT to investigate immune cell infiltration in TCMR and search candidate diagnostic markers for TCMR.

In this study, five gene expression omnibus (GEO) microarray datasets of TCMR, which contained 1561 kidney transplantation biopsy samples with stable allograft function and 224 TCMR samples were downloaded. Four datasets (GSE98320, GSE114712, GSE124203 and GSE129166) were merged into a meta-data cohort. Differentially expressed genes (DEGs) were identified between the TCMR and STA samples. Machine-learning algorithms were performed to filter diagnostic biomarkers of TCMR. The selected DEGs were validated in another validation cohort (GSE69677). Then, the proportions of immune cells in total samples based on their gene expression profiling available from public database were quantified by Cell-type identification by estimating relative subsets of RNA transcript (CIBERSPRT). Finally, the relationship between diagnostic markers and the infiltrating immune cells characteristics was approached by Spearman’s rank correlation analysis.

## Materials and Methods

### Renal Biopsy Specimens

Human specimens comprised renal biopsy specimens with the diagnosis of TCMR (biopsies from five patients as defined by The Banff Classification of Allograft Pathology) and stable function (biopsies from 5 patients with normal kidney allograft pathologic change) after kidney transplantation from Frist Affiliated Hospital of Nanjing Medical University.

### Microarray Data

Data used in this study were obtained from the public domain, The series of matrix files of GSE98320, GSE114712, GSE124203, GSE129166 and GSE69677 (**Table 1**) were identified and downloaded from the GEO database (http://www.ncbi.nlm.nih.gov/geo). GSE98320, GSE114712, GSE124203 and GSE129166 were merged into a metadata cohort after preprocess and removing the batch effect by “SVA” package of R software (21). GSE69677 was used as the validation cohort. All samples were collected from allograft after kidney transplantation.

**Table 1:** Basic information of the microarray datasets from GEO.

GEO datasets						
	GEO No.	Platform	Species	TCMR	STA	References
Meta-data cohort	GSE98320	GPL15207	Homo sapiens	81	774	(16)
	GSE114712	GPL10558	Homo sapiens	4	8	(17)
	GSE124203	GPL13667	Homo sapiens	138	566	(18)
	GSE129166	GPL570	Homo sapiens	1	61	(19)
Validation cohort	GSE69677	GPL14951	Homo sapiens	24	52	(20)

### DEG Screening and Functional Enrichment Analysis

The “limma” package of R (http://www.bioconductor.org/) was used for background correction, normalization between arrays, and differently expressed genes (DEGs) selection with Cut-off criteria of the adjusted *P* value (false discovery rate, FDR) < 0.05 and |log fold change (FC)| > 1. To analyze the function of DEGs, gene ontology (GO) analysis and Kyoto Encyclopedia of Genes and Genomes (KEGG) pathway analysis were conducted by “clusterProfiler” package. FDR< 0.05 was considered statistically significant.

### Candidate Diagnostic Biomarker Screening

Hubgenes were selected with degrees ≥10 in http://string-db.org. The least absolute shrinkage and selection operator (LASSO) is a regression-based algorithm that uses regularization to improve the prediction accuracy. The LASSO regression algorithm performed by “glmnet” package was used to predict the genes associated with TCMR and STA samples.

### Diagnostic Value of Biomarkers in TCMR

The ROC curves were generated to investigate the predictive value of the identified genes. The area under the ROC curve (AUC) value was calculated to determine the diagnostic effectiveness in discriminating TCMR from STA samples and further verified in GSE69677.

### Discovery of Immune Cell Subtypes

CIBERSOR, which is a computational algorithm for quantifying cell fractions from tissue gene expression profiles, was used to calculate immune cell infiltrations in this study. The putative abundance of immune cells was estimated using a reference set with 22 types of immune cell subtypes (LM22) with 1000 permutations (22). Spearman’s rank correlation analysis performed by R package “corrplot” and “ggplot2” was used to explore the association with each other.

### Statistical Analysis

Continuous variables were analyzed by Student’s t-test and Mann-Whitney U-test. LASSO regression analysis was performed by the “glmnet” package. ROC curves were used to assess the sensitivity and specificity of the biomarkers. The spearman correlation test was used to estimate the relationship between biomarkers and infiltrating immune cells. All statical analyses were performed by R (version 4.0.3). The results with *P* < 0.05 were considered statistically significant.

### Immunohistochemical Staining

Formalin-fixed kidney tissue sections (3 µm) obtained from human kidney biopsy specimens were deparaffinized, hydrated, and antigen-retrieved. The antibodies [anti‐CXCR6 (ab273116; 1;500; Abcam, USA) and anti‐CXCL13 (ab246518; 1:1000; Abcam, USA)] were incubated overnight at 4°C after sections blocked with 5% normal goat serum. The stained slides were photographed using a Nikon Eclipse 80i microscope equipped with a digital camera (DS-Ri1, Nikon, Shanghai, China).

## Results

### Identification of DEGs in TCMR

**Figure 1:**
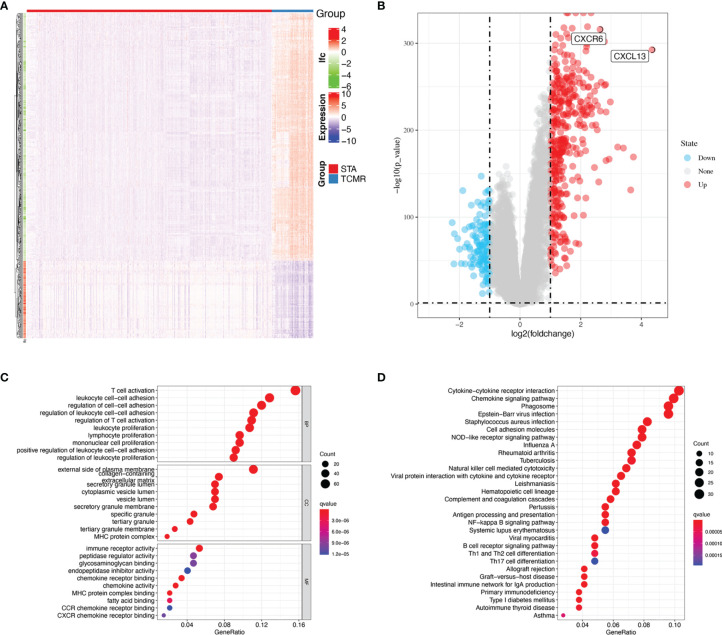
Functional enrichment analysis of DEGs. **(A)** Heatmap for DEGs between TCMR and STA group. **(B)** Volcano plot for DEGs. **(C)** KEGG analysis on DEGs. **(D)** GO analysis in DEGs.

As shown in **Table 1**, a total of 224 TCMR and 1561 STA samples from four GEO databases (GSE98320, GSE114712, GSE124203 and GSE129166) were retrospectively analyzed in this study. All data were analyzed with limma package after the batch effects were removed. The heat map for DEGs in meta-data cohort was shown in **Figure 1A**. Totally 129 upregulated DEGs and 378 downregulated DEGs were identified in **Figure 1B**.

### Functional Enrichment Analysis

To extract the key terms and pathways that share close biological associations with TCMR, GO and KEGG analysis were carried out by using a computational framework. Top 30 enriched GO and KEGG terms were shown in **Figures 1C, D**. The GO results demonstrated that DEGs were mainly associated with T cell activation, lymphocyte proliferation, mononuclear cell proliferation, MHC protein complex and immune receptor activity. The KEGG results indicated that the enriched pathways mainly involved natural killer cell mediated cytotoxicity, antigen processing and presentation, B cell receptor signaling pathway, allograft rejection and graft-versus-host disease. Those conclusions suggested that immune regulation is closely bound up with TCMR.

### Identification and Validation of Diagnostic Biomarkers

**Figure 2:**
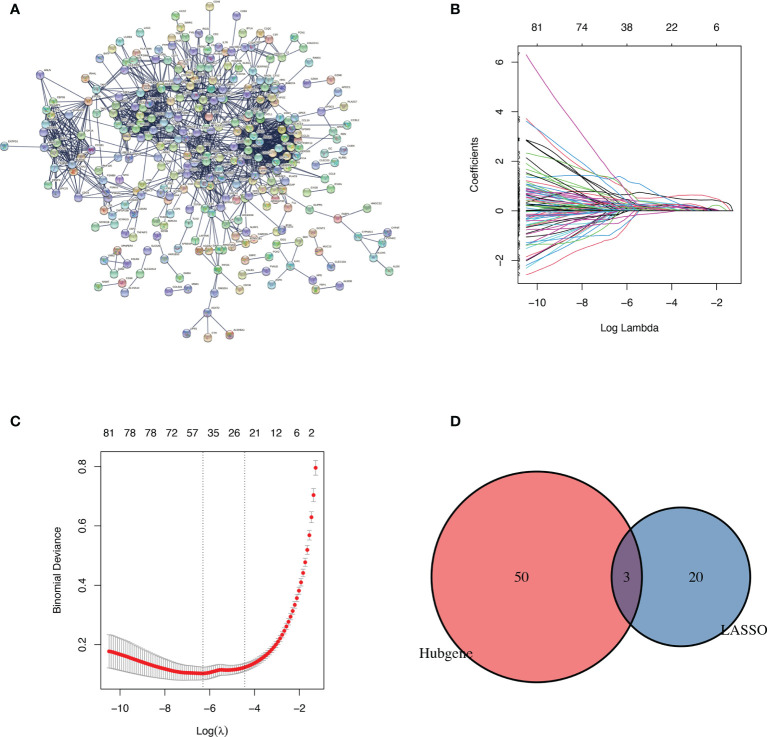
Identification of diagnostic markers. **(A)** Protein-protein interaction network of DEGs. **(B, C)** identification of biomarkers using the LASSO regression algorithm. **(D)** Venn diagram demonstrating three markers shared by PPI network and the LASSO regression algorithm.

**Figure 3:**
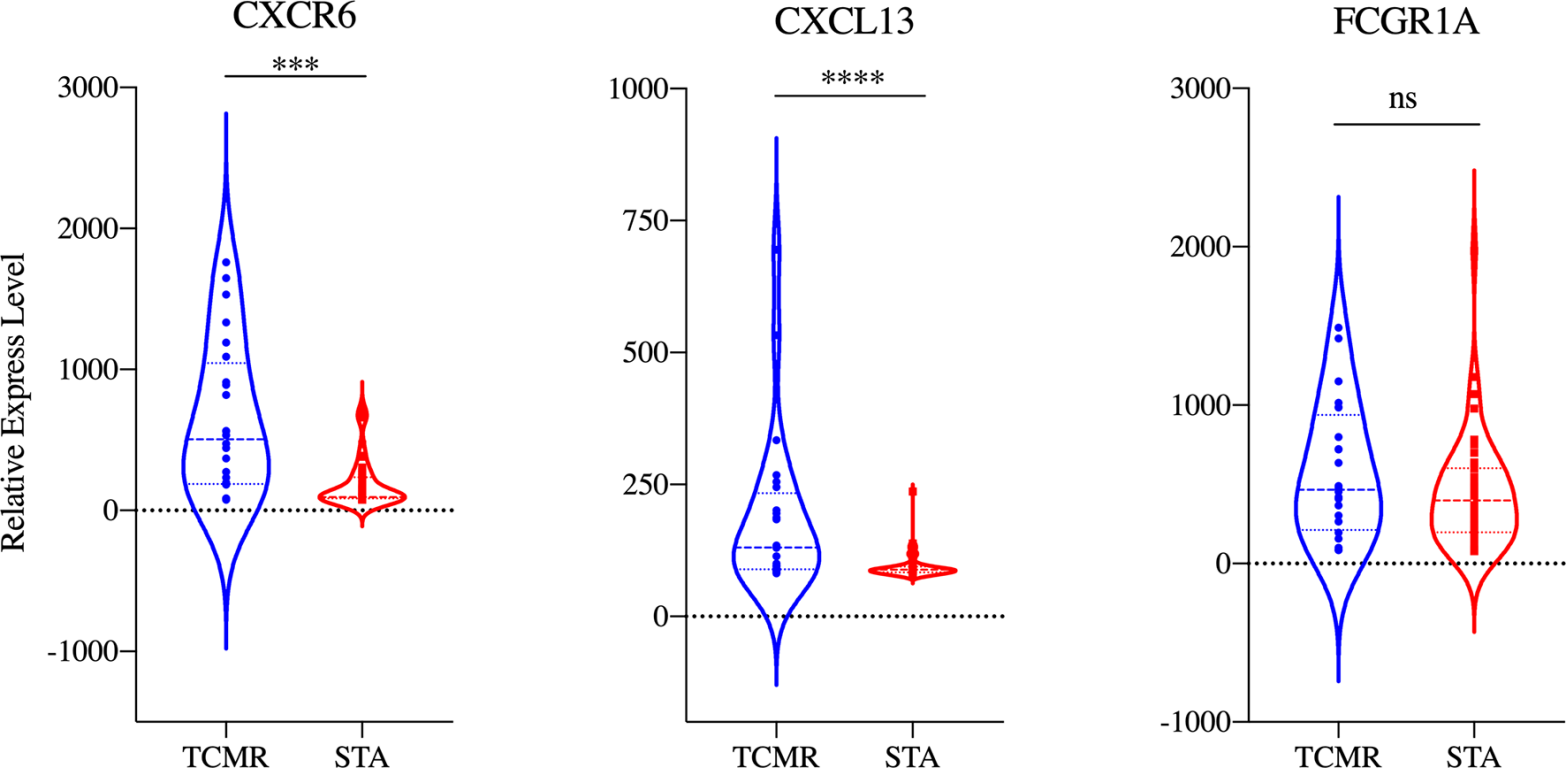
Validation of the expression of the three diagnostic biomarkers in the GSE69677 dataset. ***p < 0.001; ****p < 0.0001.

A PPI network comprised of 506 nodes and 1491 edges was created after removing independent nodes. 53 genes were chosen for the next analysis by calculation (**Figure 2A**). The DEGs were subsequently narrowed down using the LASSO regression algorithm. As shown in **Figures 2B, C**, 23 variables were selected as diagnostic biomarkers for TCMR. The intersection of the two algorithms contains three overlapping genes (CXCR6, CXCL13 and FCGR1A) (**Figure 2D**). Moreover, the expression of the three genes were verified by GSE69677. The result showed a significantly higher expression levels of CXCR6 and CXCL13 in TCMR group than STA group (**Figure 3**). However, there was no significantly difference in expression of FCGR1A between the two groups.

### Diagnostic Efficacy of Biomarkers in TCMR

**Figure 4:**
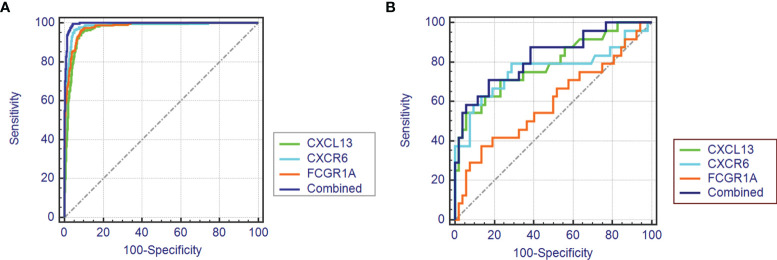
The receiver operating characteristic (ROC) curve of the diagnostic effectiveness of the three diagnostic markers. **(A)** ROC curve of CXCR6, CXCL13 and FCGR1A in the metadata cohort. **(B)** ROC curve of CXCR6, CXCL13 and FCGR1A in the GSE69677 dataset.

As shown in **Figure 4A**, the diagnostic ability of the three biomarkers in discriminating TCMR from the STA samples demonstrated a favorable diagnostic value, with an AUC of 0.981 (95%CI 0.974-0.987) in CXCR6, AUC of 0.968 (95%CI 0.958-0.976) in CXCL13 and AUC of 0.978 (95%CI 0.969-0.984) in FCGR1A. When combined together, the diagnostic ability in terms of AUC was 0.995 (95%CI 0.990-0.998) in the meta-data cohort. Moreover, to confirm the discrimination ability of the three DEGs, the AUC in CXCL13 was 0.788 (95%CI 0.679-0.873), the AUC in CXCR6 was 0.761 (95%CI 0.649-0.851) and the AUC in FCGR1A was 0.594 (95%CI 0.475-0.705). The diagnostic ability of three biomarkers combined yielded an AUC of 0.825 (95%CI 0.720-0.902) (**Figure 4B**). Considering the AUC value of FCGR1A in validation cohort and the similar expression levels of FCGR1A in two groups, only CXCL13 and CXCR6 were included in the next analysis.

### Immune Cell Infiltration

**Figure 5:**
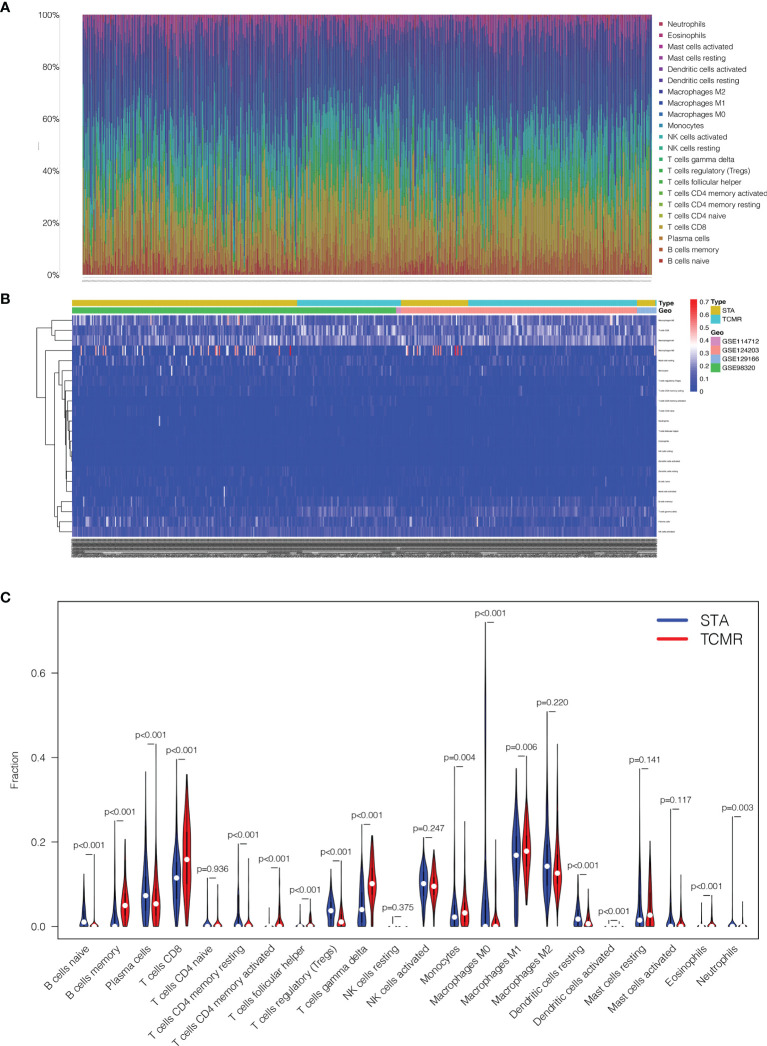
Comparisons of 22 important immune fractions between TCMR and STA groups. **(A)** The Specific 22 immune fractions represented by various colors in each sample were shown in the barplot. **(B)** Distributions of immune cells in TCMR and STA samples in the heatmap. **(C)** Comparison of 22 immune cell subtypes between TCMR and STA groups.

22 types of immune cells in kidney transplantation samples were included in infiltration calculation by the CIBERSORT algorithm. All the 224 TCMR and 1561 STA samples were screened according to the criterion CIBERSORT *P* < 0.05. The infiltration results were exported as a bar plot and heat map in **Figures 5A, B**.

Next, the composition of immune cells in TCMR and STA samples were analyzed. The proportions of naive B cells (*p* < 0.001), plasma cells (*p* < 0.001), CD4^+^ memory resting T cells (*p* < 0.001), regulatory T cells (*p* < 0.001), M0 macrophages (*p* < 0.001), resting dendritic cells (*p* < 0.001) and neutrophils (*p* = 0.003) in TCMR samples were significantly lower than those in STA samples. Meanwhile, the proportions of memory B cells (*p* < 0.001), CD8^+^ T cells (*p* < 0.001), CD4^+^ memory activated T cells (*p* < 0.001), follicular helper T cells (*p* < 0.001), gamma delta T cells (*p* < 0.001), monocytes (*p* = 0.004), M1 macrophages (*p* = 0.006), activated dendritic cells (*p* < 0.001) and eosinophils (*p* < 0.001) in TCMR samples were significantly higher than those in STA samples (**Figure 5C**).

### Correlation Analysis Between the Two Biomarkers and Infiltrating Immune Cells

**Figure 6:**
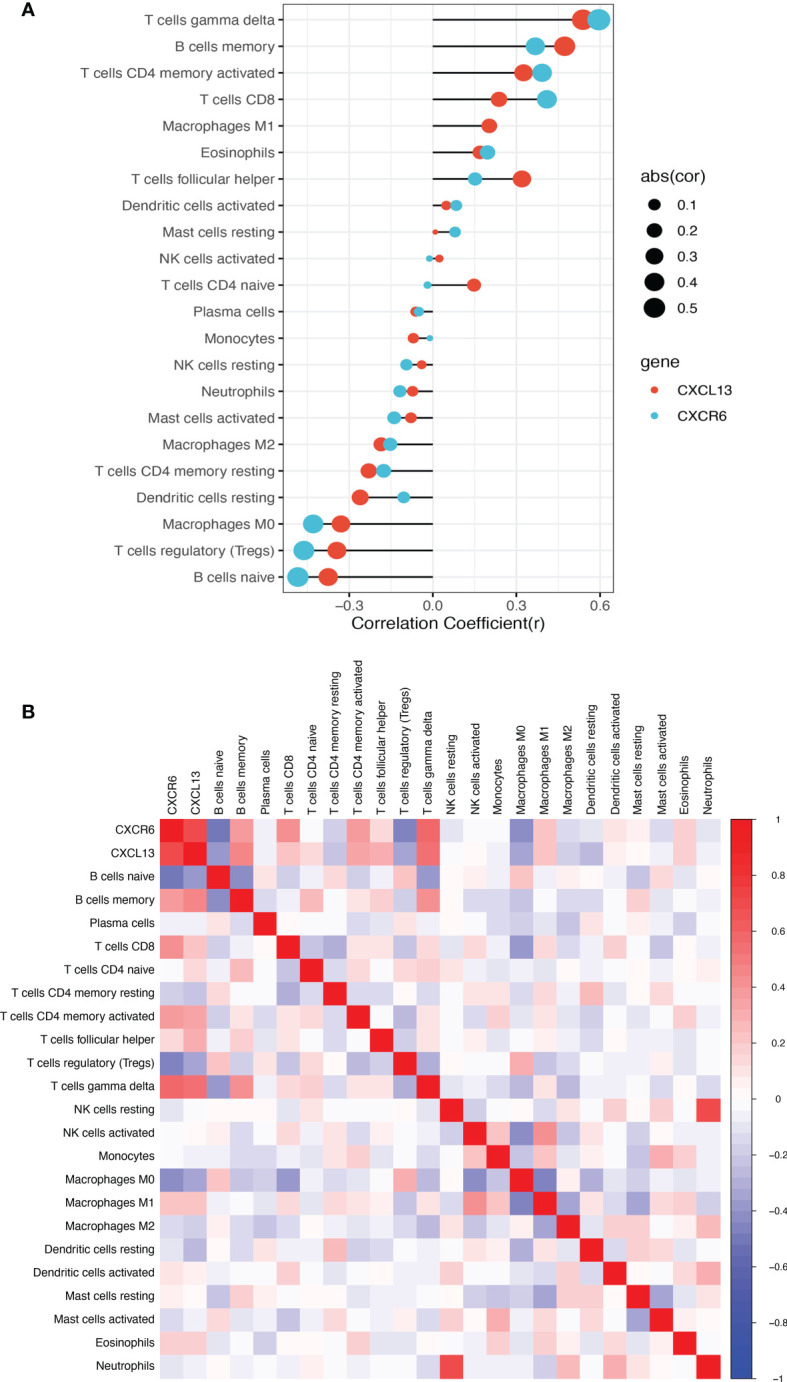
The co-expression patterns among fractions of immune cells. **(A)** Correlation between the two biomarkers and infiltrating immune cells in TCMR. **(B)** Correlation matrix of all 22 immune cell subtype compositions and the two biomarkers. Both horizontal and vertical axes demonstrate immune cell subtypes. Immune cell subtype compositions (higher, lower, and same correlation levels are displayed in red, blue, and white, respectively).

As shown in **Figures 6A, B**, both CXCR6 and CXCL13 were positively correlated with gamma delta T cells (*p* < 0.001), CD4^+^ memory activated T cells (*p* < 0.001), CD8^+^ T cells (*p* < 0.001) and M1 macrophages (*p* = 0.006), and negatively correlated with M2 macrophages (*p* < 0.001) and regulatory T cells (*p* < 0.001).

### Overexpression of CXCR6 and CXCL13 in TCMR Samples

**Figure 7:**
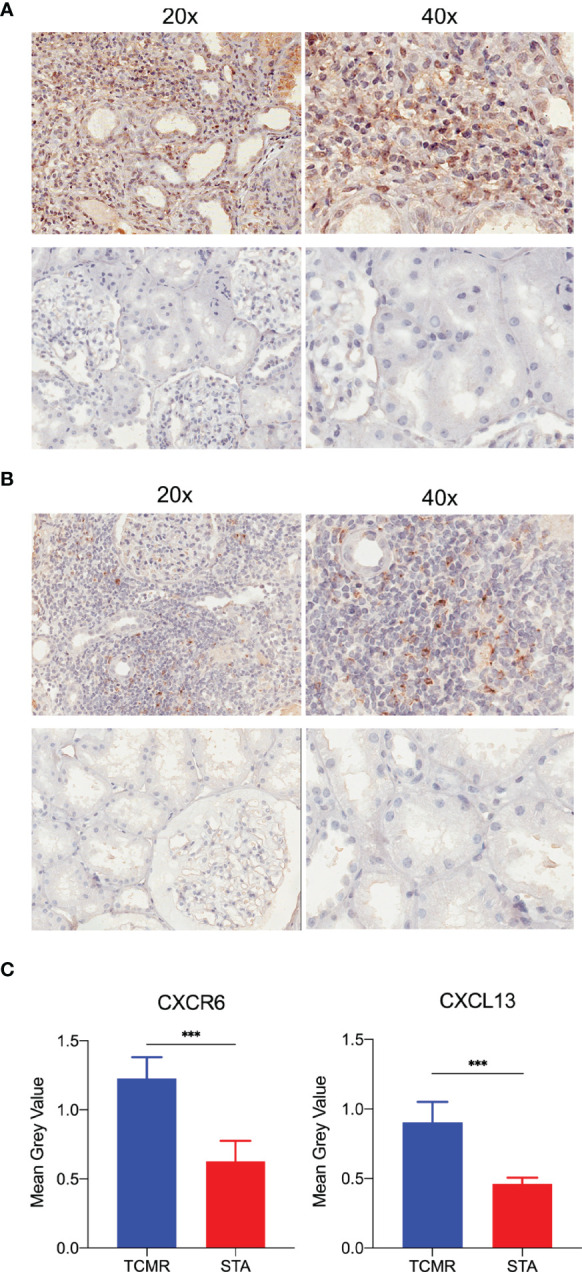
The immunohistochemistry staining and image analysis of CXCR6 and CXCL13 in human renal biopsy samples. **(A)** Immunohistochemistry staining of CXCR6 in TCMR and STA samples. **(B)** Immunohistochemistry staining of CXCL13 in TCMR and STA samples. **(C)** Semi-quantitative analysis of CXCR6 and CXCL13 in immunohistochemistry staining by mean grey value. ***p < 0.001.

To testified expression levels of CXCR6 and CXCL13 in human kidney allografts, five TCMR and five stable function biopsy samples was detected by immunohistochemical staining and image analysis. In **Figures 7A, B**, both CXCR6 and CXCL13 were positive expressed in TCMR samples, but not in STA samples. In **Figure 7C**, the expression levels of CXCR6 and CXCL13 was calculated by mean grey value. in TCMR samples, mean grey values of CXCR6 (*P* = 0.0002) and CXCL13 (*p* < 0.001) were significantly higher.

## Discussion

T cell-mediated alloimmune responses have been the target for most immunosuppression drug development in transplantation. TCMR is less frequent with contemporary immunosuppression, but most patients after kidney transplantation are still facing the complications from immunosuppressant, like infection, tumors, drug toxicity and so on. The identification of biomarkers for TCMR may provide some new diagnosis and therapy choices to recipients. Halloran et al. tried to make a diagnostic standard for TCMR by a microarray-based test in a reference set of 403 TCMR biopsies in 2013 and 2016 (23, 24). Khatri et al. identified a common rejection module consisting 11 overexpressed gene in acute rejection (25). Datasets used in the module were from kidney, lung, heart and liver transplant patients. However, to improve the specificity of results, only kidney transplantation datasets were included in the present study. Molecular biomarkers could improve diagnostic and prognostic ability and could guide treatment of TCMR (26). Through GO and KEGG analysis, this study aimed to improve the susceptibility and supplement new biomarkers for TCMR diagnosis in a larger sample size. Meanwhile, our study is focused on the association between DEGs and immune infiltration in TCMR and STA tissues, which could provide a better understanding of molecular involvement in TCMR.

Four datasets from GEO were merged into one meta-data cohort. Totally 129 upregulated DEGs and 378 downregulated DEGs were identified. The GO results indicated that the DEGs were enriched in T cell activation, leukocyte proliferation, mononuclear cell proliferation and MHC protein complex. T cell activation is one of the key components of allograft rejection progress, directly mediating rejection and GVHD (27). Activated T cells express surface cytokines, leads to the activation of leukocyte proliferation pathway (28). The process causes the further inflammatory cell infiltration. Meanwhile, MHC protein activation was suggested an independent risk factors for chronic allograft rejection (29). The KEGG results indicated that the DEGs were enriched in multiple Autoimmune diseases and T-cell subtypes. Those diseases are closely related to autoimmune disorders (30–34). S Romagnani found that TH1 cells were involved in contact dermatitis, organ-specific autoimmunity and allograft rejection. At the same time, TH2 cells are responsible for the initiation of the allergic cascade (35). As we known, there is dynamic equilibrium between TH17 cells and Tregs. The balance of the two adjusts immune response in allograft rejection (36). Besides, the DEGs were enriched in GVHD and allograft rejection, which only occur in transplantation. These indicated that immune factors played an important role in allograft rejection, which confirmed our findings in the GO and KEGG analysis.

Based on PPI network and lasso regression, CXCR6 and CXCL13 were identified. CXCR6, which is the receptor of CXCL16, expresses on activated CD8^+^ T cells, type 1-polarized CD4^+^, and constitutively expresses on NKT cells (37, 38). Additionally, CXCL16/CXCR6 has been shown causing the activation of extracellular signal-regulated kinase (ERK) mitogen-activated protein kinase (MAPK) and Akt/protein kinase B (PKB) pathways. Germanov et al. have identified the critical roles for CXCL16/CXCR6 in NKT cells activation and the regulation of NKT cells homeostasis (39). Jiang et al. showed that expression of CXCR6 was upregulated on CD8^+^ T cells by transplant rejection (40). Thus, CXCR6 is involved in immune response to tumor, infection and allograft rejection. CXCL13 was the first identification of a B-cell specific chemokine, and it termed B Cell-Attracting Chemokine 1. Dysregulation of the CXCL13 affecting both B cells and T follicular helper cells function was a major player in autoimmune disorders (41). Lena Schiffer et al. suggested that CXCL13 could serve as a relevant circulating biomarker to identify B-cell involvement in kidney transplant recipients with TCMR and relevant B-cell involvement (42). The majority of acute rejections are T cell-mediated. However, intra-graft B-cell accumulation also plays an important role in T cell-mediated rejection and correlates with a worse outcome (43–45). CXCL13 might hinder tumor progression by increasing the number of immune cells at the tumor site which was evidenced by its correlation with greater prognosis and survival in multiple tumor types (46). Therefore, the two biomarkers belonged to the CXC family have confirmed participating in immune infiltration of multiple diseases.

Infiltration of 22 familiar immune cells subtypes in TCMR and STA samples was calculated by the CIBERSORT algorithm. As a result, subtypes which were significantly different in two groups were identified. In retrospective studies, TCMR in kidney was defined by interstitial infiltrate, tubulitis and intimal arteritis (47). The cells involved in interstitial infiltration included T cells, macrophages and dendritic cells. T cells in the infiltration were CD4^+^ and CD8^+^ effector and effector memory cells (48). Under the CIBERSORT algorithm, the distribution of immune cells subtypes in this study were consistent with those results. CD8^+^ T cells (*p* < 0.001), CD4^+^ memory activated T cells (*p* < 0.001) and activated dendritic cells (*p* < 0.001) were significantly higher in TCMR group. Tregs could inhibit the function of activated CD4^+^ and CD8^+^ T cells, B cells, macrophages, and dendritic cells in allografts. Tregs suppressed both autoimmune and alloimmune responses and were particularly effective in protecting allografts (49). The proportion of Tregs (*p* < 0.001) was significantly lower in TCMR samples. Both CXCR6 and CXCL13 were negatively correlated with Tregs (*p* < 0.001).

Besides, the expression differences of two biomarkers in human allograft were still needed verification. By immunohistochemical staining and image analysis, the expression levels of CXCR6 and CXCL13 were verified significantly higher in TCMR samples. Identification of stable diagnostic biomarkers is always the purpose of research. Although multiple biomarkers have been found, our study hoped to minimize the bias of statistical analysis and identified biomarkers through larger sample size and different algorithms. The two biomarkers met the requirements.

The limitations of this study should be acknowledged. Firstly, this study is retrospective and lacks clinical data for TCMR patients. Secondly, to ensure the accuracy of our analysis and validation, GSE69677 was chosen for verification. The sample size of GSE69677 was still small, which could be the reason why FCGR1A failed validation. In addition, the cell-types which express CXCR6 or CXCL13 are still needed confirmed by counterstaining. Meanwhile, the co-relationship and mechanism of CXCR6 and CXCL13 on occurrence and development of TCMR is still not clear. Further prospective studies with larger sample sizes should be conducted to verify our conclusions.

Taken together, bioinformatic methods were performed in this study. CXCR6 and CXCL13 were identified as diagnostic biomarkers of TCMR. CXCR6 and CXCL13 were correlated with several subtypes of immune cells in TCMR, especially Treg cells. The immunohistochemical staining and image analysis results of TCMR samples in our department are consistent with our findings. These two biomarkers have the potential to be auxiliary diagnosis markers and targets for immunotherapy in patients with TCMR.

## Data Availability Statement

Publicly available datasets were analyzed in this study. This data can be found here: https://www.ncbi.nlm.nih.gov/geo/query/acc.cgi?acc=GSE98320 https://www.ncbi.nlm.nih.gov/geo/query/acc.cgi?acc=GSE114712 https://www.ncbi.nlm.nih.gov/geo/query/acc.cgi?acc=GSE124203 https://www.ncbi.nlm.nih.gov/geo/query/acc.cgi?acc=GSE129166 https://www.ncbi.nlm.nih.gov/geo/query/acc.cgi?acc=GSE69677.

## Ethics Statement

The studies involving human participants were reviewed and approved by ethics committee of the First Affiliated Hospital of Nanjing Medical University. The patients/participants provided their written informed consent to participate in this study.

## Author Contributions

HZ, HL, and LS contributed equally to this study. HZ, HL, and LS performed the data analysis and management. HZ drafted the manuscript. ZW, MZ, DZ, and ZH contributed to the revisions of the manuscript. MG and RT designed this study and provided the project funding. All authors read and approved the final manuscript.

## Funding

This work was supported by the National Natural Science Foundation of China [grant numbers 81900684, 81870512, 81770751, 81570676, 81470981, 81100532], Project of Jiangsu Province for Important Medical Talent [grant number ZDRCA2016025], the “333 High Level Talents Project” in Jiangsu Province [grant numbers BRA2017532, BRA2016514, BRA2015469], the Standardized Diagnosis and Treatment Research Program of Key Diseases in Jiangsu Province [grant number BE2016791], the Open Project Program of Health Department of Jiangsu Province [grant number JSY-2-2016-099], Jiangsu Province Natural Science Foundation Program [grant number BK20191063].

## Conflict of Interest

The authors declare that the research was conducted in the absence of any commercial or financial relationships that could be construed as a potential conflict of interest.

## Publisher’s Note

All claims expressed in this article are solely those of the authors and do not necessarily represent those of their affiliated organizations, or those of the publisher, the editors and the reviewers. Any product that may be evaluated in this article, or claim that may be made by its manufacturer, is not guaranteed or endorsed by the publisher.

## Abbreviations

AUC, Area under the ROC curve; CIBERSORT, Cell-type identification by estimating relative subsets of RNA transcript; DEGs, Differently expressed genes; FC, Fold change; FDR, False discovery rate; GEO, Gene expression omnibus; GO, Gene ontology; KEGG, Kyoto Encyclopedia of Genes and Genomes; LASSO, Least absolute shrinkage and selection operator; STA, Stable; TCMR, T cell-mediated rejection.
